# Histamine, a vasoactive agent with vascular disrupting potential, improves tumour response by enhancing local drug delivery

**DOI:** 10.1038/sj.bjc.6603461

**Published:** 2006-11-14

**Authors:** F Brunstein, J Rens, S T van Tiel, A M M Eggermont, T L M ten Hagen

**Affiliations:** 1 Laboratory of Experimental Surgical Oncology, Erasmus MC, Department of Surgical Oncology, Daniel den Hoed Cancer Centre, Room Ee 0175, PO Box 1738-3000 DR Rotterdam, The Netherlands

**Keywords:** regional treatment, histamine, doxorubicin, TAV, soft-tissue sarcomas

## Abstract

Tumour necrosis factor (TNF)-based isolated limb perfusion (ILP) is an approved and registered treatment for sarcomas confined to the limbs in Europe since 1998, with limb salvage indexes of 76%. TNF improves drug distribution in solid tumours and secondarily destroys the tumour-associated vasculature (TAV). Here we explore the synergistic antitumour effect of another vasoactive agent, histamine (Hi), in doxorubicin (DXR)-based ILP and evaluate its antivascular effects on TAV. We used our well-established rat ILP model for *in vivo* studies looking at tumour response, drug distribution and effects on tumour vessels. *In vitro* studies explored drug interactions at cellular level on tumour cells (BN-175) and Human umbilical vein endothelial cells (HUVEC). There was a 17% partial response and a 50% arrest in tumour growth when Hi was combined to DXR, without important side effects, against 100% progressive disease with DXR alone and 29% arrest in tumour growth for Hi alone. Histology documented an increased DXR leakage in tumour tissue combined to a destruction of the TAV, when Hi was added to the ILP. *In vitro* no synergy between the drugs was observed. In conclusion, Hi is a vasoactive drug, targeting primarily the TAV and synergises with different chemotherapeutic agents.

Tumour necrosis factor (TNF)-based isolated limb perfusion (ILP) is an approved and registered treatment for sarcomas confined to the limb in Europe since 1998 and is currently carried out in approximately 30 cancer centres with referral programmes for limb salvage around the continent (Eggermont *et al*, 1996a). Isolated limb perfusion with TNF and melphalan also yields excellent antitumour effects against melanoma (Lienard *et al*, 1992) and various other tumours in the clinical setting (Bickels *et al*, 1999; Olieman *et al*, 1999; Eggermont *et al*, 2003). The mechanism of action is based on the vasoactive effects of TNF, leading to a significant enhancement of tumour-selective melphalan uptake (de Wilt *et al*, 2000) and secondarily to a complete destruction of tumour vasculature (Eggermont *et al*, 1996b).

An important drawback of the use of TNF is its highly toxic nature mandating strict monitoring of leakage to the systemic compartment during ILP. Moreover, this toxic profile of TNF limits expansion of its use to less controllable sites. Therefore, other possible vasoactive drugs were sought and tested in our preclinical rat ILP model as potential candidates (Brunstein *et al*, 2004; Hoving *et al*, 2005). In this perspective, we showed strong synergy of histamine (Hi), an inflammatory mediator, when combined to melphalan in ILP, including a 66% overall response rate (OR) with 33% complete responses (CR) (Brunstein *et al*, 2004).

The aim of this study is to evaluate the effects of Hi on TAV by means of histological studies and also explore whether the synergistic effect of Hi would also apply to the combination with doxorubicin (DXR), an important chemotherapeutic drug in solid tumour treatment (Santoro *et al*, 1995; O'Byrne and Steward, 1999). Based on the assumption that DXR is the best single agent for systemic therapy, with activity in more than 20% of the treated patients, some Italian centres use it in the TNF-based ILP instead of melphalan, with circa 26% complete histologic necrosis (Rossi *et al*, 1999, 2005). Using the experimental ILP model in rats bearing syngenic soft-tissue sarcomas, the ability of the combined treatment to improve tumour response is evaluated. The effects of Hi on endothelial cells and TAV as well as on drug distribution are evaluated *in vivo*, taking advantage of the natural fluorescence of DXR and combining different histological stainings.

## MATERIALS AND METHODS

### ILP protocol

Male inbred Brown Norway rats were obtained from Harlan-CPB (Austerlitz, the Netherlands), weighing 250–300 g and were fed a standard laboratory diet *ad libitum* (Hope Farms Woerden, the Netherlands).

Small fragments (3 mm) of the syngeneic BN-175 sarcoma were inserted subcutaneously in the right hind leg of the animals as described previously (de Wilt *et al*, 1999). Tumour growth was measured daily with a calipre and the volume was calculated using the formula 0.4(*A*^2^ × *B*) (where *B* represents the largest tumour diameter and *A* is the diameter perpendicular to it). When tumour diameter exceeded 25 mm rats were killed by cervical dislocation, under anaesthesia. At the end of the experiment all the rats were killed by the method specified.

The treatment consisted of the experimental ILP, as described previously (de Wilt *et al*, 1999). Briefly, 7–10 days after implantation, tumours reached a diameter between 12 and 15 mm and were amenable to the procedure. Under anaesthesia (intraperitoneal ketamine and intramuscular hypnomidate), the inguinal vessels were reached through an incision parallel to the inguinal ligament, canulated and connected via a roller pump to an oxygenated reservoir where drugs were added in boluses. A groin tourniquet occluded collateral vessels, warranting a proper isolation of the limb.

The 5 ml total volume perfusate consisted of: haemaccel alone (Boehring Pharma, Amsterdam, the Netherlands); haemaccel+400 *μ*g DXR (80 *μ*g ml^−1^) (Adriablastina®, Farmitalia Carlo Erba, Brussels, Belgium); haemaccel + 1000 *μ*g Hi (200 *μ*g ml^−1^) (kindly provided by Maxim Pharmaceuticals Inc., San Diego, CA, USA) or haemaccel with 400 *μ*g DXR and 1000 *μ*g of Hi.

Tumour dimensions were measured every day for volume calculation. Response was classified as: progressive disease (PD), increase of more than 25%; no change (NC), volume kept in the range of −25 to +25%; partial remission (PR), decrease between −25 to −99% or complete response (CR), no palpable tumour, initial volume as compared to volume on day 9.

Limb function was clinically observed as the ability to walk and stand on the perfused limb after ILP. On a scale from 0 to 2, grade 0 is a severely impaired function where the rat drags its hind limb; grade 1, a slightly impaired function (cannot use it in a normal way, but stand on it);and finally, grade 2 is an intact function (normal walking and standing pattern).

Locoregional toxicity was also evaluated by clinical observation of limb oedema. Oedema was defined as absent (−), or present, and in this case graded in a scale from + to ++++, according to the extent of increase in volume of the treated limb as compared to the opposite, not treated limb.

The studies were carried out in accordance with protocols approved by the Animal Care Committee of the Erasmus University Rotterdam, the Netherlands.

### Histologic evaluation after Hi-based ILP

Two animals for each group were killed by cervical dislocation directly and 24 h after ILP, and tumours and a piece of underlying muscle were excised, fixed in 4% formaldehyde solution and embedded in paraffin. The slides were stained with haematoxylin and eosin and CD-31 by the Pathology Department of the Erasmus MC. Images were taken on a Leica DM-RXA microscope supplied with a Sony 3CCD DXC camera.

### Perls iron staining – histologic evaluation after Hi-based ILP

Two animals for each group were killed by cervical dislocation 7 days after ILP, and tumours and a piece of adjacent muscle were excised, fixed in 4% formaldehyde solution and embedded in paraffin. The slides were stained by Perl's method, a qualitative technique based on the release of ferric iron from hemosiderin by acid treatment, forming ferric chloride. The ferric iron reacts with potassium ferrocyanide to form ferric ferrocyanide, an insoluble blue compound known as Prussian blue (Bancroft and Cook, 1984). Images were taken on a Leica DM-RXA microscope supplied with a Sony 3CCD DXC camera.

### DXR distribution and evaluation of vascular function

To gain insight into *in vivo* intratumoral drug distribution, three animals for DXR alone and three for Hi + DXR were submitted to standard ILP plus the addition of 20 *μ*l of the vessel staining FITC-lectin (*Bandeireae simplicifolia*, BS-I Isolectin B4, Sigma, Zwijndrecth, the Netherlands) to the perfusate. Directly after the procedure, the animals were killed by cervical dislocation, tumours were excised, snap frozen in liquid nitrogen and stored at −80°C. Thick sections of 25 *μ*m were mounted with Mowiol and evaluated by confocal microscopy with a Zeiss LSM 510 Meta (488 nm laser with 505–505 band pass filter (FITC) and 543 nm laser with 560 long-pass filter (DXR)). Nine different fields per animal were selected and photographed (three fields per slide, in a total of three slides per animal). Images were further processed, using Image Tool® for Windows 2000. First, the colours of the images were separated, for quantification of vessel density (green) and drug distribution (red). Next, images were binarised, with a lower threshold setting based on the negative control, and percentage of positive pixels was determined. Data were plotted with GraphPad Prism for Windows 2000.

### Immunohistochemistry staining antialbumin

For the evaluation of the presence of albumin extravasation into the tumour, two rats submitted to each of the following ILP: (1) sham; (2) DXR; (3) Hi or (4) Hi+DXR were included in this study. Tumours were excised, fixed in 4% formaldehyde solution and embedded in paraffin right after the ILP.

Sections were deparaffinised, rehydrated and treated with 10 mM sodium citrate buffer, pH 6.0, at 100°C in a microwave oven for 10 min. Next, the slides were washed and incubated with rabbit-anti rat albumin (Nordic Immunological Laboratories, Tilburg, the Netherlands) at 1 : 1000 in Phosphate buffered saline (PBS), overnight at 4°C. After washing, antibody binding was detected using diaminobenzidine substrate-chromogen (Sigma-Aldrich, Zwijndrecth, the Netherlands) for 5 min at room temperature. Slides were counterstained with Mayer's haematoxylin, dehydrated with ethanol, cleared with xylene and finally mounted with Entellan (Merck, Darmstadt, Germany). Images were taken on a Leica DM-RXA microscope equipped with a Sony 3CCD DXC camera.

### Martius/scarlet/blue (MSB) for connective tissue and fibrin

Two rats submitted to each of the following ILP: (1) sham, (2) DXR, (3) Hi or (4) Hi+DXR were included in this study. Tumours and underlining muscle were excised, fixed in 4% formaldehyde solution and embedded in paraffin right after the ILP. Tumour slides were first deparaffinised and hydrated before being stained by MSB method for fibrin. Shortly, slides were placed in Martius yellow (Sigma) for 2 min, rinsed with distilled water; placed in Crystal scarlet (Ponceau 6R) (Sigma) for 10 min; differentiated with Phosphotungstic acid (Sigma) until only fibrin was red (*circa* 10 min); and placed in Methyl blue (Sigma) until collagen was blue (also *circa* 10 min). Then, slides were briefly rinsed with 1% aqueous acetic acid, rapidly dehydrated with ethanol, cleared with xylene and finally mounted with Entellan (Merck). Images were taken on a Leica DM-RXA microscope equipped with a Sony 3CCD DXC camera.

### Cytotoxicity assay

Direct interaction between DXR and Hi was evaluated *in vitro* on BN-175 tumour cells and endothelial cells.

BN-175 tumour cells (isolated from the spontaneous, rapidly growing and metastasizing soft-tissue sarcoma) (Kort *et al*, 1984) were grown in RPMI-1640 essential medium (Life Technologies, Breda, the Netherlands) supplemented with 10% foetal calf serum and 0.1% penicillin–streptomycin (Life Technologies, Breda, the Netherlands).

Cells were plated 24 h before treatment in 96-well, flat-bottomed, microtitre plates (Costar, Cambridge, MA, USA) at a concentration of 10^5^ cells ml^−1^, final volume of 100 *μ*l and allowed to grow as a monolayer. Next, they were incubated at 37°C in 5% CO_2_ for 48 h in the presence of medium alone or medium plus different concentrations of DXR and Hi. Histamine ranged from 0 to 200 *μ*g ml^−1^ and DXR from 0 to 5 *μ*g ml^−1^.

Growth of tumour cells was measured using the sulphorhodamine-B (SRB) assay (Skehan *et al*, 1990). In brief, cells were washed with PBS, incubated with 10% trichloric acetic acid for 1 h at a temperature of −4°C and washed again. Cells were stained with SRB for about 15–30 min, washed with 1% acetic acid and allowed to dry. Protein-bound SRB was dissolved in TRIS (10 mM, pH 9.4). Extinction was measured at 540 nm and the percentage of growth inhibition was calculated according to the formula: percentage of tumour cell growth=(test well/control well) × 100%. The drug concentration leading to 50% reduction in absorbance, as compared to control (IC_50_), was determined from the growth curve. The experiments were repeated four times.

Human umbilical vein endothelial cells (HUVEC) were prepared by collagenase treatment of freshly obtained human umbilical veins and cultured in human endothelial–SFM/RPMI medium (Life Biotechnologies, Breda, the Netherlands) supplemented with 10% heat-inactivated human serum (Cambrex, Verviers, Belgium), 20% new born calf serum, human EGF, human bFGF and 0.1% penicillin–streptomycin (Life Technologies, the Netherlands).

Human umbilical vein endothelial cells were plated 24 h before treatment in 96-well plates at 6 × 10^4^ cells ml^−1^, total volume of 100 *μ*l and allowed to grow as a monolayer. Next, they were cultured for 48 h with Hi, in concentrations ranging from 0 to 200 *μ*g ml^−1^ and DXR from 0 to 0.5 *μ*g ml^−1^. The growth and IC_50_ were determined in the same way as for the tumour cells.

### Statistical analysis

Kruskal–Wallis and Mann–Whitney *U*-tests were used to evaluate statistical significance of the results. All statistical tests were two-sided and *P*-values less than 0.05 were considered as statistically significant. Calculations were performed on a personal computer using Prism v3.0 software (GraphPad Software Inc., San Diego, CA, USA) and SPSS v10.0 for Windows 2000.

## RESULTS

### Tumour response after ILP

**Figure 1:**
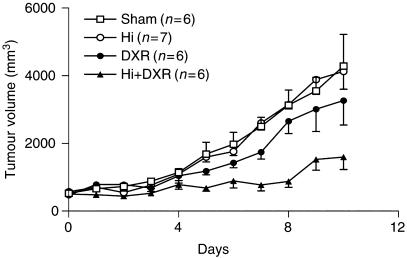
Tumour response in soft-tissue sarcoma bearing rats after Hi-based ILP. Tumor-bearing rats were submitted to ILP with sham, DXR, Hi or DXR + Hi as described in Materials and Methods. Mean tumour volumes±s.e.m. are depicted. ^*^P<0.01 on day 8 for Hi+DXR as compared to sham; *P*=0.027 on day 8 for Hi+DXR as compared to DXR alone.

While tumours grew exponentially in all rats submitted to either control or DXR alone ILP, Hi alone could arrest tumour growth for 4 days in two out of seven animals (29%). As expected, the best response was seen with the combination of Hi and DXR showing a partial regression in two animals (33%) and arrest of tumour growth for approximately 6 days in three animals (50%) (*P*<0.01 on day 8 for Hi + DXR as compared to sham; *P*=0.027 on day 8 for Hi + DXR as compared to DXR alone) (Figure 1; Table 1).

As previously seen in Hi + melphalan ILP, Hi either alone or combined with DXR did not inflict systemic side effects. As for regional toxicity, we observed a very mild (+) oedema in 17% (one out of six) of the rats treated by Hi+DXR and 14% (one out of seven) of those treated by Hi alone ILP, leading to a temporary grade 1 toxicity. The oedema compromised the limb as whole, including both the tumour and the normal tissue and was of the soft kind oedema. Isolated limb perfusion with DXR alone also caused a temporary regional toxicity in one (17%) of the treated rats scoring a grade 1 function with limb oedema lasting for 3–4 days.

### Histology

**Figure 2:**
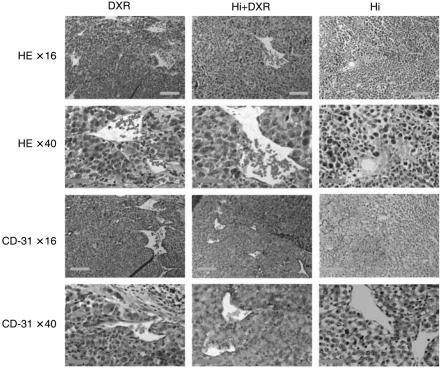
Tumour vascular destructive effect and haemorrhagic necrosis upon Hi-based ILP. Pictures of representative tumour histology (HE) and vascular destruction (CD31) right after ILP with DXR, Hi or DXR + Hi are shown. Orange bar on × 16 magnification pictures corresponds to 100 *μ*m and red bar on × 40 magnification to 50 *μ*m.

Immediately after ILP with Hi + DXR vasodilation was observed, accompanied by tumoral endothelial cell damage and haemorrhage. Next to that some oedema in the tumour was observed. Histamine alone ILP resulted in diffuse oedema, but much less haemorrhage was seen than with the combination of Hi+DXR. Sham (data not shown) or DXR alone ILP had no effect on vasodilation or haemorrhage and predominantly intact tumour cells and few necrotic spots were seen. In accordance to these findings, CD-31 staining clearly showed the Hi-mediated destruction of the TAV-associated endothelial cell lining, with a more striking effect observed in those tumours treated by the combination of Hi+DXR as compared to Hi alone ILP. DXR alone ILP clearly had no effect on endothelial cell lining (Figure 2).

### Perls iron staining – histologic evaluation after Hi-based ILP

**Figure 3:**
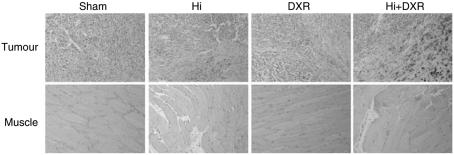
Staining by Perl's method shows Histamine-related induction of necrosis at 7 days after ILP, which is absent in muscle. Original magnification × 10. Black bar corresponds to 100 *μ*m.

In agreement with HE and CD-31 findings, Perl's method documented a haemorrhagic effect linked to Hi administration. Although DXR-alone-treated tumours had some iron deposits 7 days after ILP, mainly in the tumour tissue, these became more abundant after Hi alone ILP. After Hi+DXR ILP though, those iron deposits were clearly bigger and much more pronounced. As expected, iron deposits were mostly observed in the rim of the tumour and close to the vessels. The pattern was exactly the same as the one previously observed after Hi+melphalan ILP (data not shown). Muscle tissue from Hi-treated tumours (both with and without DXR) showed few and much smaller foci. Sham ILP had no iron deposits either in muscle or in tumour tissue. These findings further support the specific TAV-targeting action of Hi (Figure 3).

### Albumin extravasation and tumour endothelial cell matrix alterations

**Figure 4:**
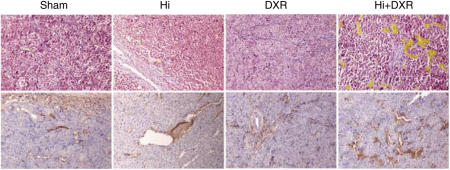
Albumin extravasation and tumor endothelial cell matrix alterations. Upper panel shows MSB staining for connective tissue and fibrin with a clear disarrangement of collagen fibres seen in tumours submitted to Hi-based ILP. Lower panel depicts most representative slides of Immunohistochemistry staining with antialbumin where albumin appears in brown, surrounding the vessels in the Hi-treated tumor tissues. More striking effects were clearly observed after Hi+DXR ILP. Original magnification × 16.

To get a better insight into the pattern of oedema an immunohistochemistry antialbumin staining was carried out. Likewise already observed in the haematoxylin and CD-31 stainings, the vessels looked dilated after Hi treatment either with or without the combination to DXR. Extravasated albumin was observed around, and close to the vessels, once more, this observation was more striking after Hi+DXR ILP (Figure 4).

Given the above findings, an additional staining specific for collagen and fibrin was carried out (MSB). Apart from the already expected Hi-related vasodilation, a very interesting observation was the Hi-related disarrangement of the collagen fibres, which could possibly play a role in improving tumour drug distribution (Figure 4). Slides from adjacent muscle showed normal anatomic structures without significant changes (data not shown), further supporting the TAV-specific effect of the treatment.

### Doxorubicin distribution and TAV evaluation by FITC–lectin ILP

**Figure 5:**
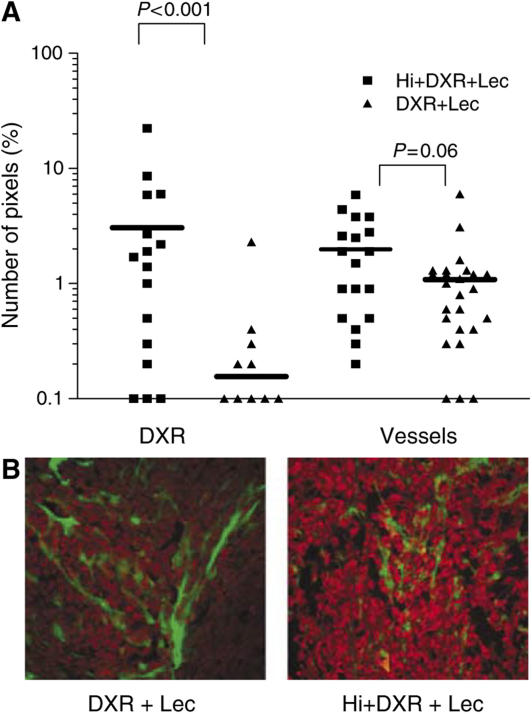
Effect of Hi-based ILP on intratumoral DXR distribution in relation to tumour vessel presence. Directly after ILP with DXR and lectin–FITC (DXR+Lec), or Histamine plus DXR and Lec (Hi+DXR+Lec) tumours were excised, frozen and 25 *μ*m thick slides were cut. Sections were examined by confocal microscopy. DXR distribution and vessel density in digital images were measured as described in Materials and Methods (**A**). Representative pictures of intratumour DXR distribution right after ILP with DXR and lectin–FITC (DXR+Lec), or histamine+DXR and Lec (Hi+DXR+Lec) are shown (**B**).

Taking advantage of the natural red fluoresence of DXR, we evaluated drug distribution within tumour and muscle by confocal microscopy of thick slides. When ILP was performed with DXR alone, some extravasation was observed around perfused (lectin-positive) vessels. Increased extravasation of DXR was seen around tumour vessels, when Hi was coadministered, while in muscle no major effects were observed. Moreover, some areas of DXR leakage could be observed in the tumour, with diffuse or even absent lectin staining, indicating severe damage to the endothelial lining of the tumour vasculature. These observations suggest an increased leakage specifically from the tumour vascular bed when Hi was added to the ILP (Figure 5). This finding was confirmed by means of intratumoral drug distribution quantification carried out through pixels intensity measurement (*P*<0.001, DXR alone compared to DXR combined to Hi). The lectin staining also revealed large areas devoid of functional vessels, mainly in the tumour centre, in which no DXR could be delivered during the ILP.

### Direct cytotoxicity of histamine

**Figure 6:**
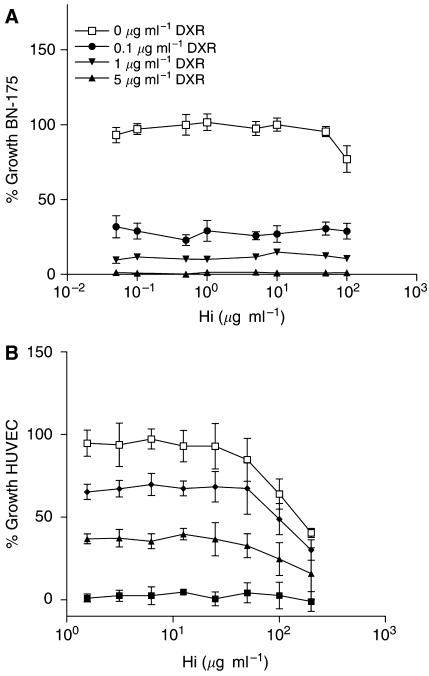
Evaluation of possible direct effects of Hi and DXR on BN-175 soft-tissue sarcoma cells and HUVEC. BN-175 tumor cells (**A**) or HUVEC (**B**) were exposed to 0–5 *μ*g ml^−1^ DXR with Hi 0–200 *μ*g ml^−1^ for 72 h. Each point represents an average of four readings. Error bars show s.d. values.

To evaluate the potential synergistic action between DXR and Hi, *in vitro* cytotoxicity assays were carried out on BN-175 tumour cells and on HUVEC. As shown in Figure 6, both agents were capable of killing endothelial cells with an IC_50_ of 200 *μ*g ml^−1^ for Hi and an IC_50_ of 0.1 *μ*g ml^−1^ for DXR. While BN-175 tumour cells were effectively killed by DXR with an IC_50_ of 0.08 *μ*g ml^−1^, hardly any effect of Hi was noticed with an IC_50_ as high as 500 *μ*g ml^−1^. Combining DXR and Hi *in vitro* had only an additive effect.

## DISCUSSION

In this study, we show, by combining the natural red fluorescence of DXR with lectin–FITC staining of functional vessels during ILP, that the vascular disrupting effect of Hi augments intratumoral delivery of DXR. Based on these observations we hypothesise that both destruction of the tumour-associated vasculature and better tumour drug distribution, when Hi was administered, added to the observed augmented tumour responses. Additional histological stainings, such as CD-31 and Perl's method, further demonstrated Hi-related endothelial lining disruption and tumour haemorrhage, respectively. It is of note that these effects were more intense in tumour than in the muscle tissue, in agreement with the previously reported four-fold increase in melphalan uptake by tumour as compared to muscle (Brunstein *et al*, 2004). Indeed, it has been long established that the susceptibility of endothelial cells to vasoactive agents vary according to their grade of differentiation. Agents such as TNF and Hi have a stimulatory effect on slowly proliferating cells from resting vessels found on normal tissue. On the other hand, fast proliferating endothelium as seen in placenta, wound tissue and tumours have an inhibitory pattern of response to vasoactive agents, and are consequently more vulnerable to haemorrhage (Denekamp, 1984; van de Wiel *et al*, 1992).

Histamine and TNF have many similarities concerning their mechanism of action in tumour response after ILP, including TAV targeting, with an increased tumour drug uptake accompanied by selective TAV destruction (Brunstein *et al*, 2004; Hoving *et al*, 2006). Furthermore, both Hi and TNF show synergistic response with chemotherapeutic drugs and both require the combination of chemotherapeutics to achieve the best tumour responses after ILP. Yet, it is of note that while a TNF-alone ILP resulted in progressive disease in all treated animals, Hi-alone ILP could arrest tumour growth in the range of 29–50% of the treated animals (this study and Brunstein *et al* (2004)).

Accordingly, *in vitro* studies disclosed a direct cytotoxic effect of Hi against tumour cells and endothelial cells (HUVEC), while TNF required the combination with interferon and peripheral blood mononuclear cells, for having an effect on endothelial cells *in vitro* (Seynhaeve *et al*, 2006).

Speculating on possible mechanisms of Hi-induced endothelial damage, it shall be taken into account that the gap formation of endothelial cells occurs via inositol phosphate second messenger and increased intracellular calcium concentration (Carson *et al*, 1989). The recovery process however, is mediated by an increased intracellular cAMP level and negative feedback inhibition, a pathway shared by ‘endothelial stabilisers’, such as prostaglandin (PGI2). Histamine stimulates PGI2 production after very short time incubation periods of 1–2 min (Resink *et al*, 1987; Wu and Baldwin, 1992) and also stimulates the production of other eicosanoids such as HETE, which can affect cell function through incorporation into membrane lipids and altering the properties of the membrane (Wang *et al*, 1990). Previous studies showed that HETE production reduces the production of PGI2 and leads to the contraction of the endothelial monolayer, finally compromising endothelial function and contributing to the atherogenic process (Revtyak and Campbell, 1992).

Another possible mechanism of Hi-related tumour endothelium damage could be, similarly to the one described to TNF, an induced congestion causing haemorrhage and oedema by impaired blood flow and finally resulting in haemorrhagic infarction. Supporting this theory are the different stainings documenting an Hi-related vasodilation and haemorrhage. It is already known for TNF that platelets and von Willebrand factor play a major role (Nooijen *et al*, 1996), yet for Hi this process still requires further investigation.

Furthermore, in this study we show that the antitumour effect of Hi in a regional therapy model was not restricted to melphalan but was also present in combination with DXR, resulting in tumour regressions or tumour growth arrest in 67% of the rats. Histamine alone arrested tumour growth in 29% of the animals, while tumour progression was observed in virtually all rats in sham or DXR alone groups.

Tumour response rates in this study were not as good as those previously reported for the combination of Hi plus melphalan. Yet, DXR alone was also less active than melphalan alone in our ILP model, with no antitumour effect (100% progressive disease) for DXR, against 17% partial response and 17% tumour growth arrest for melphalan (Brunstein *et al*, 2004). These findings are in accordance with the literature where melphalan is described as the drug of choice for ILP in most centres worldwide, with the best response rates and lower complication indexes (Thompson *et al*, 1998; de Wilt *et al*, 1999).

A possible explanation for the reduced efficacy of DXR could be its cycle dependency, while melphalan does not have this restriction. Taking into account that during an ILP drugs are delivered in a higher dosage but only once, apposed systemic chemotherapy where drugs are given repeatedly, this difference in activity might play an important role. Furthermore, the relatively poor penetration of DXR has already been reported as probably related to a rapid uptake of this drug by the perivascular cells. DXR binds avidly to the DNA within these cells, thus reducing the amount available for diffusion to distal cells (Primeau *et al*, 2005). In the case of increased permeability, due to the combination with vasoactive agents such as TNF or Hi, DXR would still not perform as well as an alkylating agent, namely melphalan. On top of this, according to the work of Takemoto *et al* (2003), the alkylating reaction with cellular DNA, resulting in the formation of DNA crosslinks would be most significantly enhanced at elevated temperatures, with a reported activation energy for melphalan of 72.4±7.9 kcal, between 37 and 41°C, which is precisely the range of temperature used in the ILP setting. These authors provided further evidence that alkylating agents are the best option for regional treatment and also elegantly showed that the drug of choice at elevated temperatures, such as those used during regional treatment, might be different from the drug of choice at the physiological temperature (Takemoto *et al*, 2003).

Specifically in the case of Hi, the batch used in this study was less active *in vitro* on both tumour and endothelial cells as compared to previous results (Brunstein *et al*, 2004), and possibly resulted in a partial loss of the direct effect of Hi towards tumour and endothelial cells *in vivo*. Strikingly, tumour endothelial lining destruction and haemorrhage remained similar after Hi-based ILP, quite comparable to previous results. This observation was well documented by the different histologic stainings used.

An additional important finding of our study is the Hi-related disarrangement of the collagen fibres, which we speculate could play an additional role in improving tumour drug distribution. Further studies, for a better evaluation and understanding of this important step, in improving tumour response rates with the use of vasoactive drugs are underway.

In conclusion, the inflammatory mediator Hi acts as a vasoactive drug, targeting the tumour-associated vasculature and is capable of synergising with different chemotherapeutic agents. More importantly, this occurred without systemic side effects and only a mild regional toxicity, with a temporary limb oedema, completely reversible after 3–4 days. These findings support a potential role of Hi in regional treatment and organ perfusions in the clinic.

## Figures and Tables

**Table 1:** Response in BN-175 soft tissue sarcoma-bearing rats after doxorubicin-based ILP in combination with Histamine over a total period of 08 days

**Treatment^a^**	**CR^b^**	**PR (%)**	**NC (%)**	**PD (%)**
Sham (*n*=5)	—	—	—	100
DXR (*n*=6)	—	—	—	100
Histamine (*n*=7)	—	—	29	71
Hi+DXR (*n*=6)	—	17	50	33

a Doxorubicin (DXR, 400 *μ*g) and Histamine (Hi, 1000 *μ*g) were added as boluses to the perfusate (5 ml).

b Responses were scored as described in Materials and Methods. CR=complete response; PR=partial response; NC=no change; PD=progressive disease.
